# Review of Vision-Based Environmental Perception for Lower-Limb Exoskeleton Robots

**DOI:** 10.3390/biomimetics9040254

**Published:** 2024-04-22

**Authors:** Chen Wang, Zhongcai Pei, Yanan Fan, Shuang Qiu, Zhiyong Tang

**Affiliations:** School of Automation Science and Electrical Engineering, Beihang University, Beijing 100191, China; venus@buaa.edu.cn (C.W.); peizc@buaa.edu.cn (Z.P.); fyn123@buaa.edu.cn (Y.F.); zb2003108@buaa.edu.cn (S.Q.)

**Keywords:** lower-limb exoskeleton robots, computer vision, environmental perception, gait planning

## Abstract

The exoskeleton robot is a wearable electromechanical device inspired by animal exoskeletons. It combines technologies such as sensing, control, information, and mobile computing, enhancing human physical abilities and assisting in rehabilitation training. In recent years, with the development of visual sensors and deep learning, the environmental perception of exoskeletons has drawn widespread attention in the industry. Environmental perception can provide exoskeletons with a certain level of autonomous perception and decision-making ability, enhance their stability and safety in complex environments, and improve the human–machine–environment interaction loop. This paper provides a review of environmental perception and its related technologies of lower-limb exoskeleton robots. First, we briefly introduce the visual sensors and control system. Second, we analyze and summarize the key technologies of environmental perception, including related datasets, detection of critical terrains, and environment-oriented adaptive gait planning. Finally, we analyze the current factors limiting the development of exoskeleton environmental perception and propose future directions.

## 1. Introduction

Lower-limb exoskeleton robots can be classified into medical rehabilitation lower-limb exoskeletons and power-assisted lower-limb exoskeletons according to their functionality [1]. They can also be classified into medical lower-limb exoskeletons and non-medical lower-limb exoskeletons according to the end users [2]. For the control methods of different exoskeletons, exoskeletons designed for medical applications are typically implemented through predefined gaits, whereas exoskeletons designed for non-medical applications are typically implemented through motion tracking. Different control methods have different control loops that determine the role of environmental perception in the control loop.

The biggest difference between exoskeleton robots and other types of robots lies in the involvement of humans in the control loop [3]. For power-assisted lower-limb exoskeletons, the control methods aim to make the exoskeletons follow the human body movement. Therefore, accurately capturing human motion intentions is crucial. Some common control methods include Sensitivity Amplification Control (SAC) [4], direct-force feedback control [5,6,7,8], and electromyography (EMG) control [9,10,11]. SAC relies less on sensors. It treats the interaction between the user and the exoskeleton as a disturbance to the exoskeleton system. By designing a suitable control system, this disturbance can be amplified to produce a highly responsive effect of the exoskeleton on the user’s movements. Direct-force feedback control measures the interaction forces between the human body and the exoskeleton through force sensors. By controlling the magnitude of these interaction forces, the goal is to make users unable to feel the presence of exoskeletons. EMG control captures surface EMG signals through EMG sensors and then processes and analyzes them to determine the user’s motion intentions. In the aforementioned methods, both SAC and direct-force feedback control are triggered after human movement. Therefore, these methods have a certain degree of delay. EMG control can capture motion intentions before human movement. However, its application is limited by the weak generalization ability across different subjects and environments [9,10,11], and long-term use may affect user comfort and sensor accuracy. To address these problems, some studies [12,13,14,15] have applied visual sensors and related computer vision methods to exoskeletons to predict upcoming walking environments before physical interaction between the human–machine system and the environment. These works aim to achieve more accurate and robust control decisions.

Medical lower-limb exoskeletons aim to assist patients with lower-limb movement disorders to walk with gaits similar to healthy individuals. Some common control methods include methods based on human gait information acquisition [16,17,18,19,20,21], methods based on models [22,23], and methods based on Central Pattern Generators (CPGs) [24]. Control methods based on human gait information acquisition use motion capture devices (such as VICON [25], NOITOM [26], HTC VIVE kits [27], etc.) to collect gait patterns from healthy individuals, which are then used to plan the exoskeleton gaits. Control methods based on models adjust the gait to achieve stable walking using a kinematic model of the exoskeleton and the stability criterion known as the Zero-Moment Point (ZMP) [28]. Control methods based on CPGs utilize different inputs to simulate the reflexes found in organisms and the oscillations of simulated neurons, then generating periodic rhythmic signals and ultimately producing different gait patterns. These methods have been applied to quadruped robots [29] and other biomimetic robots [30]. The aforementioned control methods can achieve stable walking in specific environments but cannot achieve the independent and safe walking in unknown environments. Unlike users of power-assisted exoskeletons with complete visual-neural-muscular closed-loop motion control, for patients with lower-limb movement disorders, the visual-neural-muscular closed-loop control is incomplete [31], therefore, the motion intentions can only be transmitted to the exoskeleton through human–machine interaction methods to achieve walking in complex environments. Some common human–machine interaction methods include control panel interaction and bio-electrical signal interaction. For example, ReWalk [32,33,34] from Israel’s ReWalk Robotics Ltd. uses a control panel to select gait modes and set environmental parameters to perform daily actions such as sitting up, climbing up and down slopes, and climbing up and down stairs. Hybrid Assistive Limb (HAL) [35,36,37] from Japan’s Tsukuba University incorporates both EMG and Electroencephalogram (EEG) to detect the user’s motion intentions. The main issue with the interaction method of control panels is that it relies heavily on the user’s active participation, while the interaction method of bio-electrical signals may lead to discomfort for the user and poor generalization ability. Moreover, neither method can accurately obtain key geometric parameters in the environment, such as step width and height, for input to the exoskeletons. For medical rehabilitation exoskeletons, we hope to obtain specific terrain and geometric parameters in advance before stepping on a certain ground, providing accurate parameterized information for exoskeleton decision making and planning. Therefore, the introduction of visual signals is crucial.

**Figure 1:**
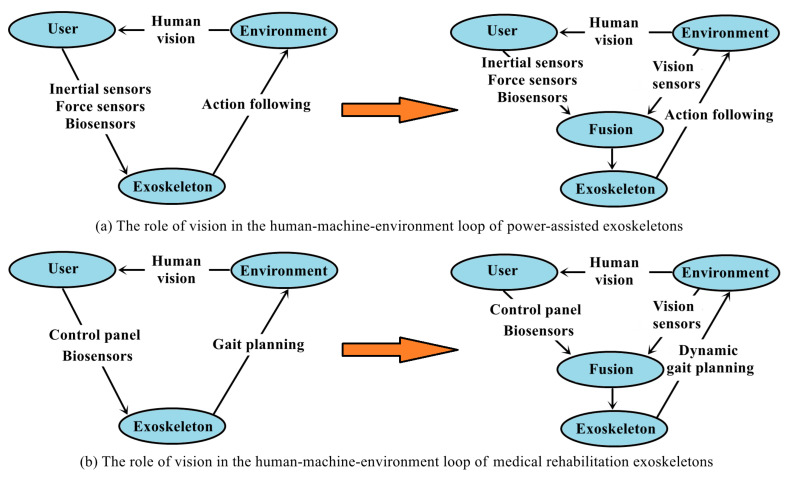
The role of vision in the human–machine–environment loop of lower-limb exoskeletons.

In summary, for power-assisted exoskeletons, the significance of incorporating vision is to anticipate the upcoming environment in advance to achieve smooth motion tracking. The perception system focuses on the classification of the overall environment, especially the environmental transition. For medical rehabilitation exoskeletons, the significance of incorporating vision is to extract key geometric features and parameters of the terrain and use these parameters for online gait planning so that the human–machine system can pass through various terrains safely and reliably, as shown in Figure 1.

The main contributions of this paper are as follows. In recent years, with the development of computer vision and deep learning, as well as the continuous decline in the cost of visual sensors and edge computing devices, it has become possible to deploy a visual system with low power consumption and high computing power on a compact wearable device like the exoskeleton. Research in this area is burgeoning but still lacks a comprehensive review of exoskeleton environmental perception systems. This paper takes exoskeleton environmental perception as the starting point and focuses on introducing the related perception algorithms after deep learning has taken a dominant position in computer vision. The main contents include related software and hardware platforms and their key technologies, aiming to provide a reference for researchers to quickly understand the current development and related issues of exoskeleton environmental perception. Furthermore, this paper also points out directions for future work by analyzing the limiting factors.

The organization of this paper is as follows. Section 2 introduces the visual sensors and control system used for exoskeleton environmental perception. Section 3 discusses the key technologies of environmental perception, including related datasets, environment classification, stair detection, ramp detection, obstacle detection and environment-oriented adaptive gait planning. Section 4 addresses the current limiting factors in exoskeleton environmental perception and proposes future directions for development. Section 5 summarizes the whole paper.

## 2. Visual Sensors and Control System

### 2.1. Visual Sensors

For wearable devices, the visual sensors installed should consider the following factors: (1) size, weight, and power consumption; (2) sensor performance, including the detection range, frame rate, accuracy, field of view, etc.; (3) robustness and compatibility; and (4) cost.

Suitable visual sensors should be small and lightweight [38] and have low power consumption. Lightweight and low-power sensors are crucial for reducing the overall burden and power consumption of the system. The sensor’s performance should meet the operational conditions of lower-limb exoskeletons in daily urban environments and be able to operate stably under different lighting conditions such as day and night. In terms of software, the Application Programming Interface (API) provided by the sensors should be widely compatible with mainstream edge computing platforms and programming languages. Moreover, since the high cost of exoskeleton devices is always a major factor restricting their widespread adoption, visual sensors should have a lower cost to control the overall expenses.

Visual sensors can be divided into passive sensors and active sensors depending on whether they emit an energy source into the environment [39]. Passive sensors, such as RGB cameras, operate under visible light conditions and mainly have two imaging methods: Charge-Coupled Devices (CCDs) and Complementary Metal-Oxide Semiconductors (CMOSs) [40]. With the rapid development of the internet, computers, and related technologies, RGB cameras have been widely used in various aspects of production and daily life, offering advantages such as low cost, compact size, and high resolution. However, RGB cameras are susceptible to lighting conditions, and their monocular vision characteristic means they cannot capture depth information from the environment, which limits their application in lower-limb exoskeletons. To enable RGB cameras to obtain depth information, binocular RGB cameras and multi-lens RGB cameras have been developed. They work on the principle of triangulation, capturing the same object from different viewpoints and calculating depth based on the disparity of the object in different images [41]. However, the triangulation of these cameras relies on feature matching between different images, which is susceptible to lighting conditions and object surface textures, therefore, they cannot achieve stable distance measurements.

Active sensors measure distance by emitting signals into the environment and sensing the reflected signals. Common active sensors include structured light cameras and Time-of-Flight (ToF) cameras. Structured light camera actively projects a pattern onto the object to be measured, which is then captured by an infrared camera. The distance to the object is calculated using triangulation. Structured light cameras are less affected by lighting conditions and textures compared to stereo cameras and offer better accuracy. However, they can be affected by reflections from smooth surfaces or interference from strong light sources. ToF cameras calculate distance by measuring the time it takes light to travel back and forth. They have the advantages of wide measurement range and high precision. However, they often have a larger size and higher power consumption, and they can be affected by multiple reflections. A common implementation of ToF technology is Light Detection and Ranging (LiDAR), which uses a rotating photosensitive diode to obtain a panoramic view of the environment [42]. LiDAR has a high resolution and strong resistance to active interference. With the development of autonomous driving and quadruped robots, the size and power consumption of LiDAR have decreased, making it one of the most popular choices for wearable device vision sensors.

**Figure 2:**
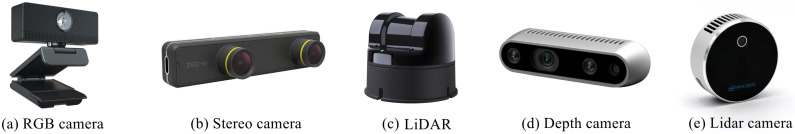
Common visual sensors: (**a**) Philips’ RGB network camera [46]; (**b**) ZED’s binocular stereo vision camera, the ZED Mini Stereo Camera [47]; (**c**) Unitree’s LiDAR L1 [48]; (**d**) Realsense’s depth camera, the D435i [49]; (**e**) Realsense’s LiDAR camera, the L515 [45].

Active sensors can easily obtain depth information from the environment but lack texture information. To take advantage of multi-modal information, active sensors have been combined with RGB cameras to make RGB-D depth cameras. For example, Intel’s RealSense depth cameras [43] and Microsoft’s Kinect depth cameras [44]. They typically use structured light for depth measurement and integrate Inertial Measurement Units (IMUs), which is beneficial for Integrated Product Development (IPD). Some RGB-D cameras use Micro-Electro-Mechanical System (MEMS) LiDAR for depth measurement, such as the RealSense L515 [45]. Some common visual sensors are shown in Figure 2.

Regarding the installation positions for visual sensors, common installation positions in previous studies include the head [50,51,52], chest [14,53,54], waist [55,56,57,58], lower limbs [59,60], and feet [61]. The advantages and disadvantages of these installation positions are shown in Table 1.

It can be seen that different assistive devices have different suitable installation positions. For lower-limb exoskeleton robots, the most suitable installation positions are the chest and waist. These positions provide a stable field of view that synchronizes with the user’s movement direction. Although there may be a certain gap between the user’s actual field of view and the movement direction, these installation positions ensure the stable operation of the environmental perception system and reduce the risk of falling.

### 2.2. Control System

To achieve an accurate perception of complex and unfamiliar environments, an appropriate control system is also needed to process the environmental information obtained from visual sensors and convert it into instructions for controlling motor movements. Currently, the common control system used for intelligent wearable devices typically consists of three layers, with each layer’s controller responsible for executing different tasks [38].

The high-level controller is responsible for acquiring and processing information from all sensors to predict the expected movement activities. For example, the high-level controller can extract environmental geometric features from images captured by visual sensors. It can estimate the system’s state through sensors, such as angle sensors, foot-pressure sensors, and IMUs. It can also capture human motion intentions through bio-electrical signals and control panels. Since the high-level controller needs to process and analyze different types of information, it usually requires high computing power and power consumption. With the development of deep learning, GPUs have appeared in some high-level controllers to efficiently run convolutional neural networks (CNNs), such as NVIDIA’s Jetson [62] from America, Raspberry Pi [63] from England, and Huawei’s Atlas 200 DK developer kit [64] from China.

After the high-level controller completes motion prediction, the middle-level controller is responsible for generating the corresponding kinematic models. For example, in medical rehabilitation exoskeletons, the middle-level controller can simulate the motion trajectories of healthy individuals or manipulate the trajectories of individual joints based on the information obtained from the high-level controller. Typically, the middle-level controller requires high real-time performance to ensure that motion commands are promptly transmitted to the low-level controller. There are some low-cost middle-level controllers, such as STMicroelectronics’ STM32 micro-controllers [65] and the Arduino micro-controllers developed by the Massimo Banzitu team [66].

**Figure 3:**
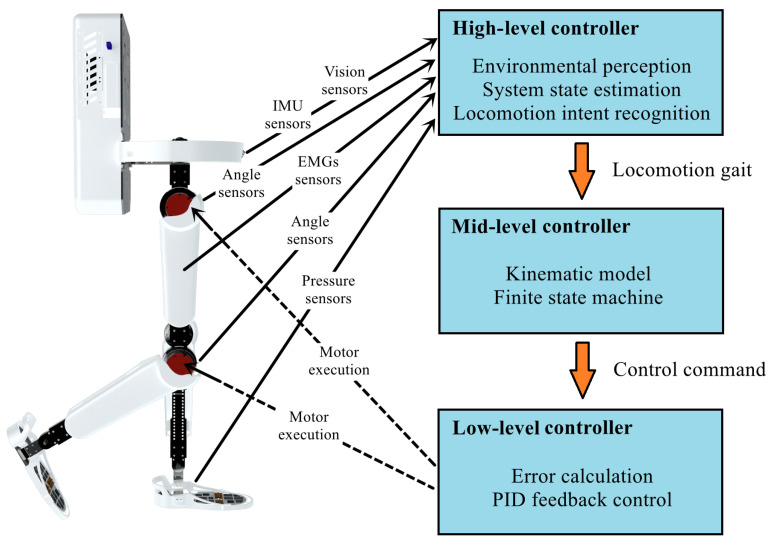
The role and relationship of controllers at different levels.

The low-level controller, also known as the motor driver, is often manufactured and integrated inside the motor itself. A typical low-level controller applies the Proportional–Integral–Derivative (PID) algorithm to calculate the deviation between the actual value and the desired value. It adjusts the position, velocity, and torque of the specified joint to form a closed-loop feedback control. The role and relationship of controllers at different levels are shown in Figure 3.

## 3. Key Technology Analysis

This section introduces the key technologies for lower-limb exoskeleton environmental perception, including related datasets, environment classification, stair and ramp detection, obstacle detection, and environment-oriented gait planning. Environment classification focuses on the overall perception of the surrounding environment, whereas the detection of stairs, ramps, and obstacles emphasizes the geometric parameter estimation of specific terrains in daily urban environments. For gait-planning methods, we mainly discuss online gait-planning methods that incorporate terrain parameters.

### 3.1. Related Datasets

Deep learning is a data-driven technology, and dataset construction is a prerequisite for researching exoskeleton environmental perception. Currently, most datasets are used for environment classification. The most popular classification dataset is the ExoNet dataset [53], which was built by Laschowski et al. This dataset consists of 922,790 images and 12 hierarchical annotations, with nine classes representing transitional scenarios where the motion pattern needs to be switched. The images in the dataset were captured using an iPhone XS Max attached to the chest with a resolution of 1280 × 720. Based on the ExoNet dataset, Kurbis and Laschowski built a dedicated dataset for stair recognition [67]. It contains 51,500 images and four classes: level-ground, level-ground to incline-stairs, incline-stairs and incline-stairs to level-ground. The images were re-annotated to improve the accuracy of the transition points between different classes. Khalili et al. [13] selected 30,000 RGB images from the ExoNet dataset and manually divided them into three classes including incline-stairs, decline-stairs and level-ground to enhance the distinguish ability between the classes. Zhu [68] built an RGB-D dataset for the environment classification of soft exoskeletons. It contains 7000 RGB-D image pairs and seven classes: grassland, road, sidewalk, incline-stairs, incline-ramps, decline-stairs and decline-ramps. Compared to RGB datasets, RGB-D datasets provide images from two modalities, which can improve the algorithm’s performance in complex scenes and scenes with poor lighting conditions to some extent.

For stair detection, some works [69,70,71] divide stair-line detection into two steps. First, mature object detection methods are applied to locate the region of interest (ROI) containing stairs in the image. Then, other methods are applied to extract the stair lines within the ROI. As a result, there are some datasets available for stair object detection. Some works [72,73,74] directly detect stair lines and stair surfaces in the entire image using a fully CNN. Consequently, there are specialized datasets for stair detection. For ramp detection, ramps are typically abstracted as planes with a certain angle relative to the ground, which have distinct geometric features. Their geometric parameters can be easily obtained without deep learning methods [75,76,77]. Therefore, there are currently no specific datasets available for ramp detection.

For obstacle detection, related algorithms should focus on objects fixed on the ground and objects that may be placed on the ground in indoor and outdoor environments. These objects also exist in universal datasets for object detection and semantic segmentation, such as the PASCAL dataset [78], COCO dataset [79], ADE20K dataset [80], NYUV2 dataset [81], and SUN RGB-D dataset [82]. Therefore, some works directly use existing datasets or re-annotated them to meet the requirements of exoskeleton environmental perception. For example, Xue [75] directly used the ADE20K dataset to train a semantic segmentation model for detecting obstacles and other terrains. Ren [83] annotated the NYUV2 dataset with support relations to achieve scene understanding based on support force analysis. Since the original annotations are relatively diverse and may not fully cover the actual operating scenes of exoskeletons, some works have built their own object detection datasets. For example, An et al. [84] built a dataset for obstacle detection based on real walking scenes.

Some common datasets built for exoskeleton environmental perception are shown in Table 2. It can be seen that the datasets built for environment classification are often large in scale due to the outstanding contribution of ExoNet and the low cost of classification annotations. However, for object detection and semantic segmentation, due to the high cost of annotation, datasets for stair detection and obstacle detection are often small in scale, which may lead to the poor generalization ability of models, and the models may fail in unfamiliar environments. Additionally, universal datasets cannot fully meet the practical detection requirements of exoskeleton environmental perception, so it is urgent to build dedicated large-scale datasets for stair and obstacle detection, and current datasets mainly focus on the detection of specific terrains, so there is still a lack of datasets for understanding walking environments.

### 3.2. Environment Classification

As a pioneering method for environmental perception, the accuracy of environment classification directly affects the accuracy of subsequent algorithms. Specifically, for power-assisted exoskeletons, environment classification directly affects the accuracy of gait pattern switching. For medical rehabilitation exoskeletons, environment classification directly affects the estimation of environmental geometric parameters.

In recent years, with the development of computer vision technologies based on deep learning, environment classification has gradually shifted from traditional image processing methods to deep learning methods. Laschowski et al. have conducted some pioneering work in the field of environment classification for exoskeletons. They developed a computer vision and deep learning-driven environment classification system [15] based on the ExoNet dataset. They applied the TensorFlow 2.3 and Keras frameworks to build and compare 12 neural network models: EfficientNetB0 [88], InceptionV3 [89], MobileNet [90], MobileNetV2 [91], VGG16 [92], VGG19 [92], Xception [93], ResNet50 [94], ResNet101 [94], ResNet152 [94], DenseNet121 [95], DenseNet169 [95], and DenseNet201 [95]. To meet the requirements of edge computing platforms, they proposed the NetScore evaluation metric, aiming to select network models that achieve higher classification accuracy with minimal parameters and computational operations. The experimental results showed that EfficientNetB0 achieved the highest accuracy, VGG16 achieved the fastest inference speed, and MobileNetV2 achieved the highest NetScore. Kurbis and Laschowski developed a specialized environment classification system for stair recognition [67]. They used MobileNetV2 to train the model, which was pretrained on ImageNet [96] to improve accuracy. The main limitation of the method is that it may misclassify floor tiles with similar textures to stairs, and when there are fewer stair steps, it may misclassify them as ground, which reflects the limitations of monocular methods. Diamantics et al. [97] proposed a Look-Behind Fully Convolutional Network (FCN) and applied it to stair recognition. The network combined multi-scale feature extraction, depth-wise separable convolution, and residual edges, enabling real-time operation on embedded and edge devices.

To address the limitations of monocular methods, some works have studied multi-modal fusion-based methods and point cloud-based methods. Zhu [68] studied the interaction between flexible exoskeletons and natural environments. An RGB-D environment classification system was built using dual-mounted D435 depth cameras, and experiments were conducted to test three fusion methods: signal-level fusion, feature-level fusion and decision-level fusion. The experimental results showed that feature-level fusion exhibited the best performance. Zhang proposed a 3D point cloud-based method [57]. Specifically, a depth camera mounted on the waist was used to capture environmental point clouds, then the downsampled point cloud was directly classified using PointNet [98]. To obtain stable point clouds, the camera’s extrinsic parameters were obtained using an IMU rigidly attached to the camera, which was used to transform the point cloud from the camera coordinate system to the ground coordinate system. The original T-Net used for point cloud normalization was removed to obtain a directional PointNet, which has better accuracy and fewer parameters compared to the original PointNet. Krausz et al. [56] proposed a series of visual features to address the variability of bio-electrical signals that may lead to prediction errors in power-assisted exoskeletons, including Depth and Normal ROI features, optical flow features, and projection features in the sagittal plane. These visual features were combined with bio-electrical signals to predict motion intentions.

### 3.3. Stair and Ramp Detection

Stairs are common architectural structures in urban environments and are widely used for floor transitions in both indoor and outdoor constructions. The research on stair detection has a long history, and its findings have been extensively applied to devices, such as humanoid robots, exoskeleton robots, quadruped robots, and smart wheelchairs. Stairs have obvious geometric features because of construction standards. Based on the geometric feature extraction methods, stair detection methods can be categorized into line-based extraction methods and plane-based extraction methods. Ramps are also common architectural structures in urban environments and are typically used for adjusting terrain heights in outdoor constructions and providing accessible pathways for individuals with disabilities.

#### 3.3.1. Line-Based Extraction Methods

**Figure 4:**
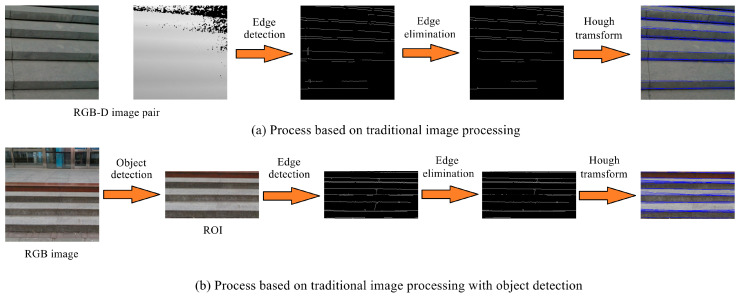
Stair-line detection methods based on traditional image processing.

Line-based extraction methods treat the geometric features of stairs as a set of continuously distributed lines [99,100]. Due to the lack of related datasets and effective feature representation methods for stair lines, traditional image processing methods have been commonly used for stair-line detection. For example, Huang et al. [101] applied Gabor filters to grayscale images to extract edges, and then short and isolated edges were removed. Stair lines were detected using the projection histograms of edge images in the horizontal and vertical directions. Similarly, Vu et al. [102] used Gabor filters to extract edges, and the Hough transform [103] was applied to detect lines. Finally, the stair lines were obtained using projective chirp transformation. Khaliluzzaman et al. [104] used a similar approach to obtain the edge image. The stair-line endpoints were then considered as the intersections of three line segments, and these intersections were extracted to obtain the geometric features of stairs. Due to the limitations of monocular vision, some works have applied depth information provided by RGB-D sensors as a supplement. For example, Wang et al. [100] first extracted a set of lines using the Sobel operator and Hough transform and then extracted one-dimensional depth features from the depth map to distinguish between stairs and pedestrian crosswalks. Khaliluzzaman et al. [105] extracted edges from both RGB and depth images, and local binary pattern features and depth features were obtained separately. Based on these features, a support vector machine (SVM) [106] was applied to determine whether stairs were present in the scene. It can be seen that traditional image processing-based stair-line detection methods generally involve extracting edges from RGB or depth images, filtering and connecting the edges, and detecting lines using the Hough transform, as shown in Figure 4a. However, these methods heavily rely on the selection of thresholds, making them unable to adapt to complex and diverse environments. In reality, they can only detect stairs in some specific scenes.

To address these problems, some works have applied mature object detection methods to stair-line detection, as shown in Figure 4b. For example, Patil et al. [69] first used YOLOV3-tiny [107] to locate the ROI containing stairs in the image. Then, traditional image processing methods were applied to extract stair lines within the ROI. Rekhawar et al. [70] applied YOLOv5 [108] to locate the ROI containing stairs, and a U-Net [109] with a ResNet34 backbone was applied to segment the stair lines within the ROI, achieving fully deep learning-based stair-line detection. Two-stage detection methods can avoid the interference of other line segments in the scene and reduce false positives. However, the real-time performance of two-stage detection methods is often difficult to ensure.

**Figure 5:**
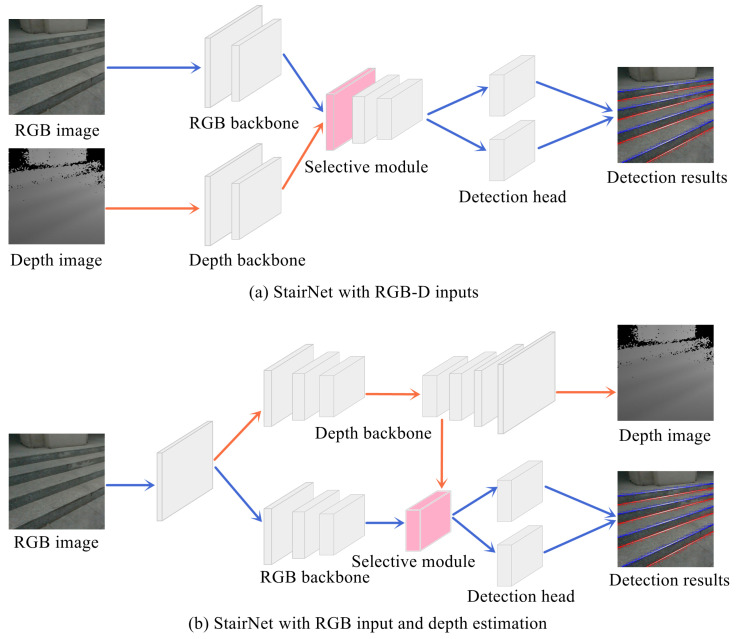
Illustration of StairNet with RGB-D inputs and StairNet with RGB input and depth estimation.

To address various problems in stair-line detection, Wang et al. proposed the StairNet series [72,73,74]. Specifically, StairNet [72] solved the problem that universal deep learning models cannot extract stair-line features directly through a novel feature representation method. It achieved end-to-end detection of stair lines with a simple fully CNN, making significant breakthroughs in both accuracy and speed. StairNetV2 [73] addressed the performance limitations of StairNet in visually fuzzy scenarios by introducing a binocular input network architecture. It also included a selective module that can explore the complementary relationship between RGB and depth images to effectively fuse RGB and depth features. StairNetV3 [74] introduced a depth estimation network architecture, aiming to balance the wide applicability of monocular StairNet and the robustness of binocular StairNetV2 in complex environments. The network architectures of the StairNet series are shown in Figure 5.

#### 3.3.2. Plane-Based Extraction Methods

Plane-based extraction methods treat the geometric features of stairs as a set of continuously distributed planes. After capturing the point clouds of environments through visual sensors, these methods apply point cloud segmentation and clustering algorithms to obtain the riser planes (vertical planes) and tread planes (horizontal planes) of the stairs. For example, Oh et al. [110] proposed a stair-plane extraction method based on supervoxel clustering. It eliminated large planar surfaces, such as walls, ceilings and floors, during the scanning process to improve real-time performance. Pérez-Yus et al. [111] proposed a stair-plane extraction method based on normal estimation. It estimated the normal and surface curvatures of each point using Principal Component Analysis (PCA) and clustered them to obtain candidate planes. The riser planes and tread planes were then extracted based on the angles between the candidate planes and the ground plane. Ye et al. [112] proposed a stair-plane extraction method based on region-growing clustering, which effectively distinguished stair planes from wall planes. It reduced the amount of point cloud data as much as possible through pass-through filtering, radius filtering and voxel filtering. Ciobanu et al. [113] proposed a stair-plane extraction method based on normal maps. They calculated the normal map from the depth map and corrected the normal map using the camera pose provided by the IMU. Then, the riser planes and tread planes were obtained through image segmentation in the normal map. This method has lower computational complexity compared to estimating normals directly from the point cloud. Holz et al. [114] proposed a fast plane segmentation method, which includes fast computation of local surface normals using integral images, point clustering in the normal space, and plane clustering in the spherical coordinate system. StairNetV3 proposed a point cloud segmentation method based on point cloud reconstruction. It transformed the point cloud segmentation problem in 3D space into a semantic segmentation problem in 2D images. Only the segmented results were reconstructed to obtain the segmented point cloud, resulting in improved real-time performance.

**Figure 6:**
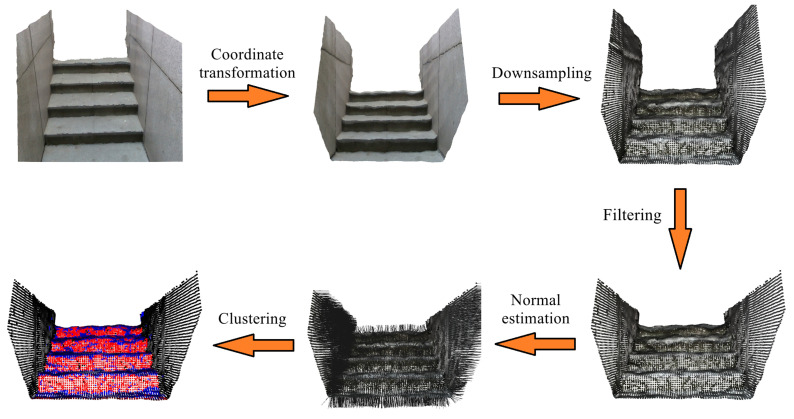
Process of plane-based stair detection methods.

In environments containing stairs, it can be seen that the method of extracting stair planes using normal information is quite effective, benefiting from the fact that the architectural structures are mostly composed of planes. The main process of these methods is shown in Figure 6. Compared to the methods of stair-line detection, the methods of stair-plane detection often perform clustering based on the normals of the point cloud. They are not affected by complex textures and lighting conditions and have better robustness. In addition, as the detection results are the riser and tread planes in three-dimensional space, it is easy to obtain the width and height of the stairs by calculating the distance between adjacent surfaces. However, compared to stair-line detection, due to the large amount of point cloud data, it is difficult to ensure the real-time performance for related algorithms on edge devices. Most works apply methods such as point cloud downsampling, dimension reduction, normal estimation optimization and clustering calculation optimization to improve real-time performance, which has been successful to some extent.

#### 3.3.3. Ramp Detection

A ramp can be considered as a plane with a certain angle relative to the ground. The key focus of ramp detection is to obtain the ramp slope to guide the corresponding gait planning. For example, Struebig et al. [76] applied the random sample consensus (RANSAC) algorithm [115] to fit the plane equation in the segmented point cloud. By searching for planes with slopes ranging from 5° to 40°, the presence of a ramp was determined. However, direct plane fitting in the segmented point cloud was computationally intensive due to the large amount of point cloud data. Therefore, some works [75,77] calculate the ramp slope by computing the projection or cross-section of the point cloud in the sagittal plane of the human body. For example, Xue [75] used the point cloud within the range of −0.3 m to 0.3 m along the x-axis to generate a binary image in the sagittal plane. Then, morphological operations and Canny edge detection [116] were applied to extract the edges from the binary image. Finally, the slope was obtained by fitting a line equation using the RANSAC algorithm. It can be seen that ramp detection requires transforming the point cloud data into the ground coordinate system, and the ramp slope can be easily calculated by determining the angle between the fitted plane equation or the line equation in the sagittal plane and the ground.

### 3.4. Obstacle Detection

Similar to autonomous driving, obstacle detection for exoskeletons needs to predict the size, position, and classification of key 3D objects near the human–machine system [117]. Accurate obstacle detection can reduce the risk of falls and instruct gait planning for crossing low obstacles. Due to the lack of related datasets, especially 3D datasets with point cloud segmentation annotations, deep learning-based 3D object detection has not yet been applied to exoskeleton obstacle detection.

Currently, mainstream methods for obstacle detection still rely on traditional point cloud segmentation. For example, Liu [118] first obtained the ground point cloud and then fitted the ground equation using the RANSAC algorithm. Then, the ground point cloud was removed, and the remaining point cloud was clustered. The cluster closest to the human–machine system was considered as the obstacle. The obstacle height was calculated using the maximum distance from the points to the fitted plane, and the obstacle width and length were calculated using the projection range of the point cloud on the ground. To reduce the computational cost of point cloud segmentation, An et al. [84] first used a CNN to determine whether the scene contained obstacles and locate the ROI containing obstacles. Then, point cloud segmentation was performed within the ROI to obtain the obstacle point cloud. Furthermore, some works detect obstacles using other methods. For example, Hua et al. [119] proposed a hybrid bounding-box search algorithm to enhance the ability to continuously cross multiple obstacles in the sagittal plane. It combined L-section tight regression and convex hull search to effectively handle interlaced obstacles with partial occlusion. Ramanathan et al. [58] detected interior holes [120] in the binarized depth map to obtain obstacles and black holes and proposed a similarity measurement method combining color, gradient direction, and 2D surface normals to distinguish obstacles from noisy artifacts.

### 3.5. Environment-Oriented Adaptive Gait Planning

Gait planning refers to the planning of the robot’s joint positions during its locomotion, typically represented as a time-angle sequence. Environment-oriented adaptive gait planning is primarily applicable to medical rehabilitation exoskeletons. This planning method incorporates environmental geometric parameters into the spatio-temporal trajectory of the exoskeleton’s end effector and calculates the joint angles through inverse kinematics.

**Figure 7:**
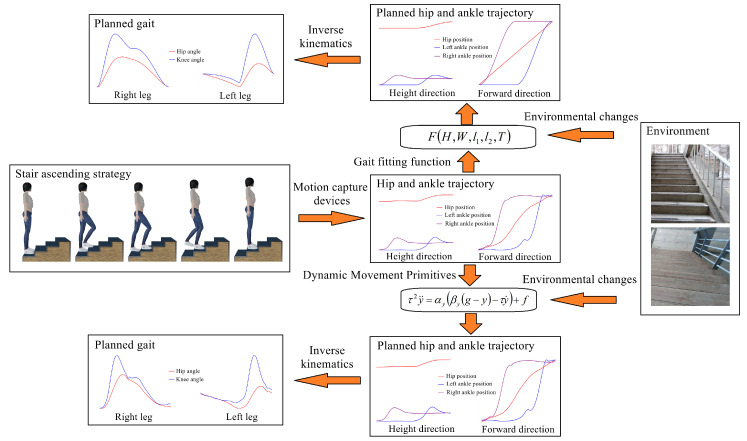
The main process of environment-oriented adaptive gait-planning methods, where $F(H,W,l_{1},l_{2},T)$ represents the fitted joint spatio-temporal domain equation, and *H* and *W* represent the width and height of the stairs, respectively. $l_{1}$ and $l_{2}$ represent the thigh length and calf length, respectively. *T* represents the gait period. $\tau^{2}\ddot{y}=\alpha_{y}\left(\beta_{y}\left(g-y\right)-\tau \dot{y}\right)+f$ represents the basic formula of a DMP. *y* represents the system status, and $\dot{y}$ and $\ddot{y}$ represent the first and second derivatives of *y*, respectively. *g* represents the target status, $\alpha_{y}$ and $\beta_{y}$ are two constants, *f* is the forcing term, and τ is the scale factor.

In practice, adaptive gait-planning methods have been developed based on gait-planning methods using walking data. Some works first collect gait data from healthy individuals ascending and descending stairs, then incorporate stair geometric parameters and human body physiological parameters as boundary conditions into the fitted trajectory. For example, Zeng et al. [121] used a fitting strategy combining polynomials and sine functions to fit the spatio-temporal sequences of the hip and ankle joints in the vertical and forward directions. Undetermined coefficients were calculated through boundary conditions, including the stair geometric parameters, thigh length, calf length, and gait period. The time-angle sequences of each joint were then calculated through inverse kinematics. Similarly, Gong et al. [122] used a fifth-degree polynomial to fit the position, velocity, and acceleration in the sagittal plane when healthy individuals walked on stairs, and the undetermined coefficients were determined based on the boundary conditions. These fitting methods are simple and practical, but when environmental parameters and users change, the trajectory may be altered, resulting in reduced biomimicry. To address this problem, [61,123] proposed a trajectory planning method based on Dynamic Movement Primitives (DMPs) [124]. DMPs can fit a target trajectory with the same trend as the source trajectory but with a different endpoint through parameter learning. They exhibit better biomimicry and are well suited for environment-oriented adaptive gait planning. However, due to the scalability of DMP-generated trajectories, the trajectory height varies with the terrain height, resulting in increased energy consumption for the human–machine system. To solve this problem, [61] optimized the generated trajectory using artificial potential fields [125,126] and biologically inspired obstacle avoidance methods [127], and [123] applied a multi-source weighted DMP approach for optimization. Taking ascending stairs as an example, the main process of environment-oriented adaptive gait-planning methods is shown in Figure 7.

Gait planning based on walking data makes the motors drive joint motion with a biomimetic gait. However, in the actual operation of the human–machine system, there are interactions between the human and the exoskeleton, which make it difficult for the exoskeleton to accurately execute the planned gait. To address this problem, some works combine gait-planning methods based on walking data with methods based on models. For example, Yu et al. [128] proposed an online correction method based on the ZMP. They first fitted the joint motion trajectory using B-spline curves, then adjusted the error between the actual and planned ZMP trajectories caused by human disturbances to improve the stability of the human–machine system. Bao et al. [52] integrated the user’s eye movement information into a Model Predictive Controller (MPC) [129] to achieve autonomous gait planning that can adjust the step length and gait period. A gait with a variable step length and gait period is crucial for gait transitions when encountering changes in terrain, as it enables the human–machine system to switch gait modes at appropriate positions before terrain transitions.

## 4. Prospects

With the continuous development of visual sensors, deep learning, and edge computing platforms, the foundation for developing environmental perception technology for lower-limb exoskeletons has been provided. However, due to the lack of related datasets, traditional image and point cloud processing methods are still the main techniques used for environmental perception at present. Inspired by autonomous driving technology, some works have started to apply deep learning computer vision methods to exoskeletons, providing them with powerful perception and autonomous decision-making abilities. However, unlike autonomous driving, the control of lower-limb exoskeleton robots should always follow the principle of the human in the loop [130]. Therefore, the research focus of environmental perception should be on how to better assist human movement. Ignoring the subjective feelings and overly relying on human control are not desirable.

In terms of environment classification, with the improvement of edge computing power and the construction of large-scale related datasets, lightweight CNNs can now be deployed on exoskeletons to provide reliable classification predictions. However, there are still some problems that need further optimization: (1) For datasets, ExoNet provides a large-scale open-source dataset of human walking environments. However, it only contains 12 classes, which cannot cover various gait-transition scenarios in daily walking environments. Additionally, the number of samples for transitional states is much smaller than that of stable states, even though transitional states should receive more attention. Similar problems exist in subsequent derivative datasets [13,67]. (2) As the triggering of environment classification precedes the triggering of EMG signals, and EMG signals precede the triggering of force and position signals, these signals have different triggering times and modalities, and their effective combination still requires further research.

In terms of stair detection, to solve the problems of traditional image processing-based stair-line detection methods and point cloud-based stair-surface segmentation methods, the StairNet series provides a deep learning-based end-to-end stair detection method. The StairNet series can quickly and accurately extract the geometric features of stairs in complex and changing environments, relying on the powerful learning ability of CNNs. However, the feature representation method proposed in StairNet results in fragmented detected stair lines, which have a different form than the original label and require post-processing algorithms to connect them. In fact, deep learning-based line detection has been successfully applied in tasks such as semantic line detection [131,132], wireframe parsing [133,134,135,136], and lane detection [137], and most related algorithms can directly obtain complete stair lines. StairNetV3 demonstrates the effectiveness of the segmented feature representation method when compared with some semantic line detection and wireframe parsing methods. However, the complete feature representation method for stair lines still needs further research. For the stair width and height estimation, plane-based detection methods offer better accuracy and stability than line-based detection methods due to the larger number of sampled points. However, the problem of real-time performance improvement has not been fundamentally solved. In future work, the method of directly obtaining point clouds of each stair step using CNNs is still worth studying.

In terms of obstacle detection, the approach used in autonomous driving is not entirely applicable to obstacle detection in exoskeletons. Autonomous driving does not require human intervention, so it is necessary to build a comprehensive scene-understanding solution to locate, measure, and track obstacles in the scene. However, for medical rehabilitation exoskeletons, we hope that humans can actively participate in various movement patterns to promote recovery. The significance of obstacle detection is to assist users in measuring the size of obstacles. For non-crossable obstacles, the exoskeleton can promptly alert the user and avoid risks, while for crossable obstacles, the exoskeleton can autonomously plan the gait based on the obstacle size to pass through smoothly. Indeed, some works [75,83] aim to provide exoskeletons with comprehensive scene-understanding capabilities. Some datasets provide complete labels for scene understanding, including 2D boxes, 2D semantic segmentation, 3D boxes, and object orientations. For example, the SUN RGB-D dataset focuses on indoor scene understanding. However, the annotation of such datasets is expensive, and there is currently a lack of datasets for understanding daily outdoor walking environments. Developing comprehensive scene-understanding abilities for exoskeletons can further enhance their intelligence and safety, but how to adjust the role of humans in the loop and construct related datasets are still urgent problems that need to be addressed.

In terms of environment-oriented adaptive gait planning, a biomimetic and online adjustable planning method remains the ultimate goal in this field. Currently, common planning methods are still mainly based on walking data, such as motion trajectory fitting, DMPs, and MPC. These methods require gait data from healthy individuals as a reference, and most works use a single source trajectory as a reference, which often leads to overfitting. For medical rehabilitation exoskeletons, the best reference trajectory is the gait of patients with lower-limb movement disorders during their healthy period, which is often difficult to obtain. Therefore, the construction of large-scale human-walking gait databases is necessary for the development of medical rehabilitation. This provides the possibility for each patient to match the best reference gait through physiological parameters such as height, weight, gender, age, etc. In addition, to ensure that medical rehabilitation exoskeletons can respond to possible emergencies like healthy individuals at any time, online adjustment methods of predefined gait are still worth further research.

## 5. Conclusions

This paper focuses on the visual perception technology of lower-limb exoskeleton robots and provides a review of the development and research status of related hardware and algorithms. We summarize the key factors and challenges that currently limit the development of environmental perception technology, aiming to provide a reference of visual perception technology for researchers in the field of lower-limb exoskeletons. We reveal the position and role of the environmental perception system in the human–machine–environment interaction loop to show the importance of visual perception. Then, we give a particular focus on the application of deep learning computer vision methods in different vision tasks. Based on the discussions of different vision tasks, we point out the current limiting factors, including the lack of scene-understanding datasets, optimization of human roles in the loop, the lack of gait databases, and look forward to the future development direction.

## Figures and Tables

**Table 1:** Advantages and disadvantages of various installation positions for visual sensors and suitable devices.

Installation Location	Advantages	Disadvantages	Suitable Devices
Head	Synchronizes with user’s view	Heavy weight may lead to discomfort and shaky images	Blind guidance equipment, upper-limb exoskeletons
Chest	The images are stable, and the view is synchronized with the movement	Camera posture is easily affected by upper-body movements	Upper-limb exoskeletons, lower-limb exoskeletons
Waist	The images are the most stable, and the view is synchronized with the movement	Low field of view, limited visual range	Lower-limb exoskeletons, lower-limb prosthetics
Lower limb	High accuracy in detecting specific terrains at close range	Restrictions on user’s lower-body dress, shaky images	Lower-limb prosthetics
Feet	High accuracy in detecting specific terrains at close range	Limited field of view, shaky images	Lower-limb prosthetics, smart shoes

**Table 2:** Some datasets for environmental perception of exoskeletons.

Source	Sensor	Number	Resolution	Annotation	Classes	Purpose
ExoNet [53]	RGB	922790	1280 × 720	Classification	12	Environment classification
Kurbis, A. G., et al. [67]	RGB	51500	1280 × 720	Classification	4	Environment classification
Khalili, M., et al. [13]	RGB	30000	1280 × 720	Classification	3	Environment classification
Laschowski, B., et al. [14]	RGB	34254	1280 × 720	Classification	3	Environment classification
Zhang, K., et al. [57]	Depth	4016	2048 Points	Classification	3	Environment classification
Zhu, H. [68]	RGB-D	7000	1280 × 720	Classification	7	Environment classification
Patil, U., et al. [69]	RGB	848	640 × 320	2D box	1	Stair detection
Rekhawar, N., et al. [70]	RGB	848	640 × 320	2D box + Stair-line mask	1	Stair detection
Habib, A., et al. [71]	RGB	510	720 × 960	2D box	2	Stair detection
Wang, C., et al. [85]	RGB	3094	512 × 512	Stair-line ends	2	Stair detection
Wang, C., et al. [86]	RGB-D	2996	512 × 512	Stair-line ends	2	Stair detection
Wang, C., et al. [87]	RGB-D	2986	512 × 512	Stair-line ends + stair-step mask	3	Stair detection
Ren, J. [83]	RGB	1449	640 × 480	Segmentation mask	13	Obstacle detection
An, D., et al. [84]	RGB-D	5000	256 × 256	2D box	2	Obstacle detection

## Data Availability

Data are contained within the article.
